# Breastfeeding in Infancy and Adult Health: A Narrative Review

**DOI:** 10.3390/children13020286

**Published:** 2026-02-19

**Authors:** Eleftherios Panteris, Ioanna Kakatsaki, Ourania Galani, Zoi Koukou, Eleftheria Hatzidaki

**Affiliations:** 1 Department of Neonatology/Neonatal Intensive Care Unit, University Hospital of Heraklion, School of Medicine, University of Crete, 70013 Heraklion, Greece; eleftherios.panteris@gmail.com (E.P.); joanne_k1991@hotmail.com (I.K.);; 2 School of Health Sciences, International Hellenic University (IHU), Sindos, 57400 Thessaloniki, Greece; koukou.zoi@ihu.gr

**Keywords:** breastfeeding, DOHaD, early-life nutrition, adult health, cardiovascular disease, adiposity, metabolic syndrome, type 2 diabetes, cancer

## Abstract

**Highlights:**

**What are the main findings?**

- Breastfeeding in infancy is consistently associated with small favourable shifts in adult cardiometabolic risk (adiposity, metabolic syndrome, type 2 diabetes) across large cohorts.
- Genetic epidemiology (including Mendelian randomisation) generally supports a modest protective association with coronary outcomes, but suggests limited mediation via lipids and substantial confounding in observational estimates.

**What are the implications of the main findings?**

- Breastfeeding should be promoted as a population-level prevention strategy with modest long-term benefits, while recognising that later-life behavioural and clinical risk factors dominate individual adult risk.
- Future studies should strengthen causal inference using triangulation (prospective cohorts, sibling/twin designs, and genetic approaches) and improve exposure ascertainment (duration, exclusivity, and neonatal intensive care feeding pathways).

**Abstract:**

Within the Developmental Origins of Health and Disease (DOHaD) framework, breast-feeding is a modifiable early postnatal exposure, but its long-term associations are difficult to separate from socioeconomic and family context. We conducted a structured literature search (PubMed/MEDLINE and Scopus; January 2015–December 2025) and prioritised large prospective/birth cohorts and genetic epidemiology studies reporting quantitative associations between breastfeeding in infancy (ever versus never, duration and, where available, exclusivity) and adult outcomes. Eighteen key primary studies were included in evidence tables across cardiometabolic, cancer, and neurocognitive domains. Overall, breastfeeding was associated with modestly lower all-cause and cardiovascular mortality, small reductions in cardiovascular disease and type 2 diabetes, and slightly more favour-able cardiometabolic profiles, including lower adiposity and higher HDL cholesterol. Where reported, effect sizes were generally small (e.g., hazard ratios typically close to 1.00), indicating limited clinical impact at the individual level but potential population relevance. Genetic analyses provide cautious support for a protective association with coronary outcomes, although lipid-mediated pathways appear to explain only a small proportion of the observed associations. Evidence for adult cancer outcomes remains mixed and largely inconclusive, while longer breastfeeding is associated with small ad-vantages in cognitive performance, educational attainment and selected psychological outcomes. Taken together, current evidence suggests that breastfeeding is associated with modestly more favourable adult cardiometabolic and neurobehavioural profiles, but its contribution to long-term health is small relative to the influence of later-life lifestyle and clinical risk factors and should therefore be interpreted cautiously.

## 1. Introduction

The first 1000 days of life represent a critical period of rapid growth and development during which environmental exposures can shape health trajectories across the lifespan [1,2]. Adverse exposures from conception through the embryonic, foetal, and neonatal stages may induce lasting structural, functional, and epigenetic adaptations that increase susceptibility to chronic diseases in adulthood [3,4,5,6]. This concept is central to the Developmental Origins of Health and Disease (DOHaD) framework, which builds on the Barker hypothesis and posits that interactions between genetic factors and early-life environmental exposures programme long-term health and susceptibility to chronic non-communicable diseases (NCDs) [7,8,9]. Although DOHaD research has largely focused on prenatal influences, the early postnatal period—particularly infancy—constitutes an additional window of vulnerability and opportunity with long-term implications for health [10,11].

Within this context, breastfeeding is widely recognised as a central modifiable postnatal exposure with the potential to extend, modify, or buffer the effects of prenatal programming. In contrast to infant formula, human milk has a distinct nutritional, hormonal, and bioactive profile, including differences in protein and lipid composition, adipokines, growth factors, immunomodulatory components, and microbiota [12,13]. Τhese elements interact to influence early growth patterns, endocrine function, immune maturation, and brain development [14,15]. Alongside these influences on early developmental pathways, infant formulas and processed baby foods may also contribute to early-life exposure to endocrine-disrupting chemicals and persistent organic pollutants, depending on product composition and contamination controls, thereby offering an additional mechanism through which infant feeding patterns could affect later cardiometabolic risk [16].

Breastfeeding has been proven to protect against morbidity and mortality throughout neonatal, infant and childhood periods, with reduced risk of respiratory infections, gastrointestinal infections, and sudden infant death syndrome, as well as favourable growth patterns. These protective effects form the basis of international recommendations for optimal infant feeding [17]. Exclusive breastfeeding for approximately the first six months of life, followed by continued breastfeeding alongside complementary foods, is endorsed by the World Health Organization (WHO) and the American Academy of Pediatrics (APA) [18,19,20]. Breastfeeding also provides partial protection against food allergy and serves as an effective non-pharmacological intervention for pain management in newborns, particularly in neonatal intensive care settings [21,22]. In addition to infant benefits, breastfeeding confers important health advantages to mothers, including reduced risks of type 2 diabetes, hypertension, and several hormone-related cancers [23].

Most research has focused on early-life outcomes, with limited evidence on the effects that appear in midlife and older adulthood, when the burden of chronic NCDs is greatest [24,25,26]. NCDs represent a major and growing global health challenge, characterised by long latency periods and progressive disease trajectories. Cardiovascular diseases, cancers, chronic respiratory diseases, and diabetes together account for the majority of NCD-related morbidity and mortality worldwide, with cardiovascular diseases alone responsible for nearly half of NCD deaths. Recent estimates indicate that NCDs now account for approximately three-quarters of all global deaths, a proportion that has increased steadily over recent decades and is projected to continue rising as populations age and survival improves [27,28].

In recent years, new studies examining adult outcomes—including cardiovascular disease, metabolic disorders, cancer, mortality, and functional health—have begun to emerge, employing diverse methodological approaches ranging from prospective cohort studies to Mendelian randomisation analyses. Although this heterogeneity complicates synthesis, it also offers novel opportunities to strengthen causal inference. The primary objective of this narrative review is to summarise and critically evaluate recent evidence on the associations between breastfeeding in infancy and health outcomes measured in adulthood, with particular emphasis on studies published between 2015 and 2025. Secondary aims are to integrate these findings within the broader life-course literature, explore potential biological mechanisms underpinning observed associations, and discuss implications for breastfeeding promotion and counselling in paediatric and primary care settings.

An important and often under-represented context is prematurity and neonatal intensive care. Many very preterm and medically complex neonates cannot be breastfed directly, they, may receive expressed maternal milk, donor human milk and/or formula, and frequently require fortification to meet nutritional needs. These early feeding pathways differ substantially from term infant feeding and may modify long-term cardiometabolic and neurodevelopmental trajectories through effects on early growth, inflammation, and the developing gut milieu. Accordingly, where available, we highlight evidence relevant to preterm birth and note this as a priority area for future life-course research.

## 2. Methodology

This review was planned as a structured narrative review, designed to integrate evidence from life-course observational epidemiology and complementary genetic approaches. We focused on adult outcomes potentially influenced by early postnatal nutrition within the DOHaD framework [13,18], while recognising that breastfeeding is socially patterned and that observational estimates are prone to confounding.

### 2.1. Search Strategy and Study Selection

A targeted search was performed in PubMed/MEDLINE and Scopus for records published between January 2015 and December 2025. Search terms combined exposure and outcome concepts (e.g., breastfeed* OR human milk OR lactation) AND (adult* OR later life OR midlife) AND (cardiovascular OR coronary OR diabetes OR metabolic syndrome OR obesity OR cancer OR cognition OR neurodevelopment OR mental health). Reference lists of key reviews and seminal cohorts were also screened to identify additional eligible primary studies and relevant mechanistic background.

### 2.2. Eligibility, Appraisal and Synthesis

We included primary quantitative studies reporting associations between breastfeeding in infancy (ever versus never, duration, and where available exclusivity) and outcomes measured in adulthood (≥18 years). Given the predominance of observational evidence, we prioritised studies with prospective ascertainment of infant feeding where available, clear and appropriately specified adjustment for socioeconomic and perinatal confounders, and robust outcome definitions (preferably registry- or clinically ascertained). We extracted the study setting, design, exposure definition, adult outcome(s), covariate adjustment set, and the main effect estimates. Because formal risk-of-bias scoring tools are not routinely applied in narrative reviews and were unlikely to be informative given heterogeneous designs and outcomes, we undertook a concise domain-based appraisal covering selection/representativeness, exposure ascertainment, confounding control, outcome ascertainment, and (where applicable) attrition/follow-up. These considerations were incorporated into the qualitative interpretation of findings, rather than used to generate a composite score. A summary of the domain-based appraisal is presented in Supplementary Table S1. Eighteen key primary studies were summarised in the evidence tables, grouped by outcome domain, alongside genetic epidemiology studies (e.g., Mendelian randomisation) when available.

## 3. Biological Mechanisms Linking Breastfeeding in Infancy to Adult Health

Breastfeeding is thought to influence adult health through several interconnected pathways acting during sensitive postnatal periods, with lasting effects on metabolism, immunity and neurodevelopment [26,29]. Human milk differs substantially from standard infant formula in macronutrient composition, bioactive factors and microbial content, and these differences are believed to shape growth, endocrine function and tissue maturation in ways that may later manifest as variation in cardiometabolic, neurocognitive and immune outcomes [30,31,32]. Within the DOHaD framework, breast milk represents a key component of the early postnatal environment, conveying nutritional, hormonal, and microbial cues that can reinforce, reshape, or mitigate signals established during foetal development [33,34].

A first pathway concerns early growth and adiposity programming. Human milk usually contains less protein than many formula preparations, which may reduce early hyperinsulinaemia and excessive adipocyte proliferation, and it provides hormones and adipokines such as leptin, adiponectin, ghrelin and insulin that may help calibrate appetite and energy balance. Higher leptin and adiponectin concentrations in breast milk have been linked to lower adiposity in infancy and early childhood, although findings are not fully consistent and often limited by sample size and residual confounding [35,36,37]. These early effects on growth and body composition are plausible intermediates between breastfeeding and adult lipid profiles, insulin sensitivity and blood pressure.

A clinically relevant intermediate phenotype worth researching is dyslipidaemia in early life, linking early-life exposures to later cardiovascular disease, and it may emerge from childhood onwards in association with adiposity and dietary patterns. While breastfeeding is not a substitute for later lifestyle and clinical risk management, early feeding practices may contribute to the developmental context in which lipid trajectories are established. This strengthens the rationale for embedding breastfeeding promotion within broader, family-centred cardiovascular prevention efforts that address childhood diet quality, physical activity and obesity prevention throughout the life course [38].

A second domain relates to immune and inflammatory regulation, including microbiome-mediated effects. Human milk contains secretory immunoglobulin A, lactoferrin, lysozyme, cytokines, chemokines and diverse oligosaccharides that support mucosal barrier integrity, modulate innate and adaptive responses and reduce pathogen adherence. [39,40,41]. These factors underlie reduced infectious morbidity in breastfed infants and are thought to contribute to modest protection against asthma, atopic dermatitis and type 1 diabetes, particularly with longer and more exclusive breastfeeding [42,43,44]. Human milk oligosaccharides (HMOs) also function as selective prebiotics that promote the growth of Bifidobacterium species and other microbial taxa associated with anti-inflammatory metabolic and immune profiles. In parallel, breastfeeding facilitates the transfer of maternal microbes from the skin, mammary gland, and entero-mammary pathway, contributing to the establishment and maturation of the infant gut microbiota during a critical window of immune and metabolic development. Resulting differences in microbiome composition, short-chain fatty acid production and systemic immune tone may shape later risks of obesity, type 2 diabetes, allergy and possibly neurodevelopmental outcomes [45,46,47].

Mode of birth is a relevant modifier and potential confounder for both breastfeeding and downstream outcomes. Vaginal birth and caesarean delivery are associated with distinct early microbial colonisation patterns, with consistent differences in initial microbiota acquisition across multiple body habitats [48]. Caesarean delivery has also been linked to lower likelihood of early breastfeeding initiation and, in some settings, shorter breastfeeding duration and greater early breastfeeding difficulty, with evidence strongest for early initiation [49,50,51]. Because early microbiome development and neurodevelopment may be jointly shaped by birth mode and feeding exposures, future studies should treat mode of birth as a key covariate and explicitly examine effect modification, as well as potential mediation pathways (e.g., breastfeeding-associated microbial taxa or metabolites stratified by birth mode), particularly in higher-risk groups such as preterm infants where gut–brain axis links are increasingly discussed [52].

Neurodevelopmental mechanisms provide a further link between breastfeeding and cognitive or mental health outcomes. Human milk supplies long-chain polyunsaturated fatty acids such as docosahexaenoic and arachidonic acids, choline and other nutrients essential for brain growth, synaptogenesis and myelination [53]. In parallel, breastfeeding typically occurs within caregiving contexts characterised by close physical contact and responsive feeding, which may promote secure attachment, stress regulation, and socio-emotional development [54]. Together, nutritional and psychosocial pathways provide a coherent framework, consistent with a DOHaD perspective, for understanding how breastfeeding as an early-life exposure may contribute modestly but meaningfully to cognitive and educational outcomes across the life course.

Epigenetic processes and life-course context likely integrate these mechanisms. Early nutrition and caregiving can modify DNA methylation, histone marks and non-coding RNA expression in genes involved in metabolic regulation, inflammation and neurodevelopment, with some changes persisting into later life [55,56,57]. Preliminary data suggest associations between breastfeeding and differential methylation at loci related to lipid metabolism, insulin signalling and immune regulation, consistent with epidemiological links to cardiometabolic and immune outcomes [58,59,60]. Chronobiotic signals in human milk, including melatonin, may represent an under-appreciated pathway through which early feeding supports neuroendocrine regulation, with recent longitudinal biomonitoring in breast milk-fed preterm infants providing supportive physiological evidence [61].

At the same time, breastfeeding practices are heavily affected by maternal education, income and broader social factors, which also influence lifestyle, healthcare access and stress across the life course [18,62,63]. This interaction between biological processes and the social environment may help explain why observed associations tend to be modest in magnitude, and provides a useful framework for interpreting mechanistic findings in relation to adult health outcomes.

## 4. Breastfeeding, Cardiovascular Outcomes and Adult Mortality

Evidence directly linking breastfeeding in infancy to adult mortality has only recently become available, despite longstanding interest in the idea that early nutrition might influence survival across the course of life [33,64].The most informative contemporary analysis is a large UK Biobank cohort in which Wang et al. examined breastfeeding history in more than 320,000 adults aged 40–73 years and followed participants for a median of about 12 years, during which over 18,000 deaths occurred. Self-reported breastfeeding in infancy was associated with a modest reduction in all-cause mortality (hazard ratio ≈ 0.95) and similar or slightly stronger inverse associations for cardiovascular and respiratory deaths after adjustment for sociodemographic, early-life and adult lifestyle factors [65]. These effect sizes are small in absolute terms but are compatible with earlier evidence [66,67] that breastfeeding is linked to more favourable cardiometabolic risk profiles, suggesting that any survival advantage is likely mediated through chronic disease rather than large differences in acute mortality.

Nakada et al. analysed over 320,000 participants and found that being breastfed was associated with a lower incidence of composite cardiovascular disease and myocardial infarction, with adjusted hazard ratios of around 0.95. These associations remained after extensive adjustment for socioeconomic indicators, birth weight and adult behaviours, although residual confounding by unmeasured aspects of early family environment cannot be excluded [68].

Li et al. extended this work in a 360,000 participant UK Biobank sample, considering a broader range of cardiovascular outcomes and intermediate phenotypes. Individuals who had been breastfed had slightly lower risks of incident cardiovascular disease, coronary heart disease and stroke, alongside more favourable levels of several cardiometabolic markers, including body mass index, waist circumference, C-reactive protein and metabolic syndrome [69]. Notably, the inverse association between breastfeeding and cardiovascular events was more pronounced among participants with higher polygenic risk scores, raising the possibility that early nutritional exposures can partially offset genetic predisposition, in line with DOHaD concepts of gene–environment interaction.

Genetic epidemiology has begun to complement these cohort studies. Zhang et al. used a two-step Mendelian randomisation design to test whether adult high-density lipoprotein (HDL) cholesterol mediates a causal pathway between being breastfed and coronary atherosclerosis, reporting that genetically predicted breastfeeding was associated with higher HDL—cholesterol, which in turn was causally related to lower coronary atherosclerosis risk [70]. Li et al. applied two-sample Mendelian randomisation to a range of cardiovascular outcomes and similarly implicated HDL-cholesterol as a partial mediator of the relationship between breastfeeding and major coronary events [71]. These analyses are appealing because they are less vulnerable to confounding by socioeconomic and behavioural factors than conventional observational designs, but they rely on strong assumptions about the validity and specificity of genetic instruments for breastfeeding and cannot capture the complexity of duration, exclusivity and timing.

Overall, the emerging evidence suggests that breastfeeding in infancy is associated with small reductions in adult cardiovascular disease and all-cause mortality. These associations are plausibly mediated through more favourable cardiometabolic profiles, including lower adiposity and higher HDL cholesterol. However, effect estimates are modest, hazard ratios are close to unity and confidence intervals in some analyses approach the null, indicating that breastfeeding is unlikely to be a major determinant of cardiovascular risk when compared with adult factors such as smoking, diet, physical inactivity, and blood pressure control [72]. From a life-course perspective, even modest shifts in risk distributions attributable to early nutrition may still be meaningful at a population level, particularly when they interact with genetic susceptibility or subsequent environmental exposures. Thus, promotion of breastfeeding for its established short- and medium-term benefits remains fully justified, while it should not be framed as a primary cardiovascular prevention strategy in adulthood. Table 1 has the relevant studies.

## 5. Adult Adiposity, Metabolic Syndrome and Diabetes

The association between breastfeeding in infancy and later adiposity has been a core focus of the long-term nutrition literature and provides important context for adult cardiometabolic outcomes. A systematic review and meta-analysis of high-quality studies from both high-income and low- or middle-income settings showed that breastfeeding was associated with a meaningful reduction in later-life metabolic risk, including an approximately 13% lower risk of overweight and obesity. These analyses indicated that these protective effects persist into young adulthood, although they are attenuated over time, with a lower prevalence of obesity observed up to around 30 years of age among individuals who were breastfed [73]. Effect sizes, however, are small, and concerns about publication bias and residual confounding have prompted calls for data in midlife and older age.

Addressing these gaps, analyses from the large UK Biobank cohort showed that adults who reported being breastfed in infancy had slightly lower body mass index and waist circumference, lower prevalence of general and central obesity, and more favourable fasting glucose and triglyceride levels than those not breastfed, after adjustment for demographic, early-life and lifestyle covariates. The prevalence of metabolic syndrome was also modestly lower (adjusted odds ratios ≈ 0.94), and C-reactive protein levels were reduced, consistent with a less adverse inflammatory profile [69]. Although these differences are small at the individual level, they are in line with earlier cohort evidence and support the notion that breastfeeding is associated with a slightly leaner body composition and more favourable metabolic markers later in life.

Complementary evidence from a nationally representative U.S. longitudinal cohort further supports these findings. Longer duration of breastfeeding in infancy was associated with lower central adiposity and reduced markers of chronic systemic inflammation in early mid-adulthood. Among adults aged 33–44 years, longer breastfeeding duration was associated with significantly lower waist circumference and C-reactive protein levels, with similar but weaker associations observed for interleukin-6. Mediation analyses suggested that central adiposity accounted for a substantial proportion of the association between breastfeeding duration and inflammation, while sibling-comparison and fixed-effects models yielded directionally consistent but less precise estimates [74].

Beyond adiposity, several studies have examined glucose–insulin homeostasis and type 2 diabetes. Meta-analytic data indicated that ever having been breastfed was associated with a lower risk of type 2 diabetes in adulthood, with pooled relative risks around 0.65–0.75, although between-study heterogeneity was considerable and many cohorts relied on self-reported diagnoses [73]. The UK Biobank analysis by Li et al. did not report incident type 2 diabetes separately, but the observed differences in fasting glucose and metabolic syndrome components were consistent with this broader picture [69].

Direct evidence on type 2 diabetes incidence from the UK Biobank was provided by Hu et al., who examined 364,562 adults free of diabetes at baseline. Over a median follow-up of 12 years, ever having been breastfed was associated with a modest but statistically significant reduction in incident type 2 diabetes compared with never being breastfed (HR ≈ 0.94, 95% CI 0.89–0.99). Notably, breastfeeding status modified the association between a type 2 diabetes genetic risk score and disease risk on both multiplicative and additive scales, with stronger genetic effects observed among those never breastfed and the combination of high genetic risk and no breastfeeding conferring more than additive risk [75]. Together, these findings suggest that early-life nutrition may partially mitigate the long-term impact of unfavourable genetic risk on type 2 diabetes development.

Breastfeeding is socially ingrained, and families who breastfeed differ from those who do not in behaviours and circumstances that influence diet, physical activity and healthcare access across the life course; residual confounding is therefore likely, even with extensive adjustment. Many studies rely on retrospective anecdotal recall of breastfeeding decades later, often without precise information on duration or exclusivity, which may attenuate true associations and obscure dose–response relations. Effect sizes are consistently modest, and most variation in adult adiposity and diabetes risk is explained by later-life exposures and genetic susceptibility.

Taken together, the evidence supports the view that breastfeeding forms part of a broader cluster of early-life factors that shift population distributions of adiposity and metabolic risk in a favourable direction. Table 2 has the latest relevant studies.

## 6. Breastfeeding and Cancer in Adulthood

In contrast to the extensive literature on cardiometabolic outcomes, evidence on whether being breastfed in infancy influences cancer risk in adulthood is relatively sparse and heterogeneous. It is important to distinguish this question from the far better-established association between maternal breastfeeding and reduced risk of several cancers in women. Large pooled analyses and meta-analyses consistently show that, among parous women, breastfeeding is associated with lower risks of breast and endometrial cancer, with dose–response relations for duration and, in some studies, for exclusivity [76,77,78,79]. These maternal benefits form a key part of the rationale for breastfeeding promotion and are not in question in the present review.

Large-scale cohort and case–control studies published in the past ten years reported heterogeneous associations between being breastfed and adult cancer risk, with no consistent evidence of a strong protective or harmful effect on overall cancer incidence [80]. One of the largest prospective investigations of adult cancer risk in relation to being breastfed in infancy was the Million Women Study in the United Kingdom. In this cohort of more than 500,000 women born between 1935 and 1950, 72% reported having been breastfed, with recall supported by good agreement with contemporaneous records in a subsample [80]. During follow-up, breastfeeding in infancy was not clearly associated with overall cancer risk, breast cancer or most other major cancer sites after multivariable adjustment. An exception was colorectal cancer, for which women who had been breastfed showed a modestly higher risk (relative risk 1.18, 95% confidence interval 1.12–1.24), accompanied by similar elevations for benign colorectal polyps and appendicitis, leading the authors to speculate about subtle long-term effects on the gastrointestinal tract while emphasising the potential for residual confounding [80].

Hameiri-Bowen and colleagues extended this line of work using UK Biobank data and including both women and men. Among more than 330,000 adults, being breastfed was associated with a very small increase in overall cancer risk in women (hazard ratio 1.05, 95% confidence interval 1.01–1.09) but not in men. In site-specific exploratory analyses, breastfed women had slightly higher risks of pre- and postmenopausal breast cancer and ovarian cancer, and breastfed men had a lower risk of oesophageal cancer; however, most of these findings did not remain statistically significant after correction for multiple testing, and the authors explicitly cautioned against interpreting them as evidence that breastfeeding is harmful [81].

Colorectal cancer has attracted particular attention because of the Million Women Study signal and broader concern about rising early-onset colorectal cancer in high-income settings [82]. In the Nurses’ Health Study and Nurses’ Health Study II, women who had been breastfed had a modestly higher risk of colorectal cancer (multivariable hazard ratio around 1.23) and of advanced colorectal adenomas, with some suggestion of stronger associations for early-onset disease before age 55 years [83]. A Japanese case–control study by Minami et al. did not demonstrate a clear overall association between being breastfed and colorectal cancer, but subgroup analyses suggested higher risks in breastfed women born after 1950 and lower risks of benign colorectal tumours in breastfed men of the same birth cohort, albeit with wide confidence intervals [84].

For other adult cancer sites, the available evidence is limited but largely reassuring. Neither the Million Women Study nor the Japanese and US cohorts have demonstrated consistent associations between having been breastfed and lung, endometrial, pancreatic or non-Hodgkin lymphoma incidence after adjustment for smoking and other lifestyle risk factors [81,83,84]. The indication of a lower risk of oesophageal cancer among breastfed men in UK Biobank is noteworthy but requires replication, particularly given the dominant role of adult exposures such as tobacco, alcohol and obesity in oesophageal carcinogenesis.

Taken together, current data do not support a major protective effect of being breastfed in infancy on total adult cancer incidence, nor do they provide robust evidence of clinically important harm. Associations observed for colorectal cancer and a small number of hormone-related cancers are modest in magnitude, often close to null, and vulnerable to residual confounding, exposure misclassification and multiple testing. Table 3 summarises the main large-scale studies that have examined being breastfed in infancy and cancer outcomes in adult offspring.

## 7. Breastfeeding and Neurocognitive, Mental Health and Human Capital Adult Outcomes

Beyond somatic health, a substantial body of research has examined the association between breastfeeding and neurocognitive development, psychological outcomes and indicators of human capital, such as educational attainment and income. Long-term birth cohort studies are particularly valuable for understanding these effects across the life course. Evidence from the Pelotas birth cohort in Brazil provides detailed insight into the long-term consequences of breastfeeding from infancy into adulthood [63]. In the 1982 Pelotas birth cohort, Victora and colleagues reported that longer breastfeeding duration was associated with higher IQ at 30 years, greater educational attainment, and higher monthly income, with a clear dose–response relationship across breastfeeding duration categories, even after adjustment for socioeconomic and perinatal factors. Individuals who were breastfed for 12 months or longer had IQ scores approximately 3.8 points higher, nearly one additional year of schooling, and around 20% higher income than those breastfed for less than one month. Mediation analyses suggested that higher IQ explained a substantial proportion of the association between breastfeeding and income. Although residual confounding cannot be entirely excluded, these findings provide rare evidence from a middle-income setting that breastfeeding may contribute to human capital formation and socio-economic mobility [63].

More recently, an econometric analysis by Khudri and Hussey used longitudinal survey data to estimate the long-term impacts of breastfeeding on adults’ educational attainment, applying instrumental-variable techniques and fixed-effects models to better account for unobserved family-level confounding. Their findings indicated that being breastfed was associated with increased likelihood of high school completion and tertiary education, with effect sizes of similar magnitude to those observed in cohort studies, reinforcing the view that breastfeeding may make a small but measurable contribution to educational trajectories in adulthood [85].

Personality traits and mental health outcomes have also been investigated, albeit less extensively and with more heterogeneous findings. In a large US cohort, Sutin and colleagues reported that adults who had been breastfed in infancy scored lower on neuroticism and higher on agreeableness and openness in the Five-Factor Model of personality, and were less likely to report lifetime diagnoses of anxiety disorders, even after adjustment for maternal education, birth weight and early socioeconomic indicators. These associations were modest but suggested that breastfeeding might be linked to more adaptive emotional and interpersonal functioning in adulthood, potentially via early differences in stress regulation, attachment security and caregiving practices [86].

In a follow-up of 3657 adults, de Mola et al. examined common mental disorders, major depression, generalised anxiety disorder and social anxiety disorder in relation to breastfeeding duration, using validated instruments and extensive adjustment for early-life confounders. Breastfeeding for ≥6 months was associated with lower odds of more severe depressive symptoms in adulthood (OR ≈ 0.69, 95% CI 0.53–0.89), with similar but less precise trends for major depression and common mental disorders, whereas no clear associations were observed for generalised or social anxiety. Overall, the study suggests a small, potentially protective association of longer breastfeeding with depressive symptom severity in adulthood, but does not support a consistent effect across all adult mental health outcomes [87].

Neuroimaging studies, although still largely confined to childhood and adolescence [88,89], have identified structural brain differences that may help explain the cognitive and personality differences reported in adulthood. Using data from the Brazilian High-Risk Cohort for Mental Conditions, Grevet et al. conducted an 8-year longitudinal MRI study of 670 children and adolescents (1326 scans) to examine how breastfeeding duration relates to brain structural development from childhood to early adulthood. Longer breastfeeding duration was associated with greater global cortical thickness in both hemispheres and with a more favourable developmental trajectory of total intracranial volume, after adjustment for multiple confounders, whereas no association was observed with cortical surface area [90]. While these findings cannot be directly extrapolated to a mature adult brain structure and function, they are consistent with a life-course model in which early nutritional and relational environments shape neural architecture with potential implications for later cognitive performance, personality and mental health.

At the same time, several methodological challenges exist in the interpretation of these associations. First, parental cognitive ability and home learning environment are powerful determinants of offspring cognitive outcomes and educational attainment, and they are also correlated with breastfeeding practices, meaning that even sophisticated statistical adjustment may not fully remove confounding [91,92]. Second, most studies rely on retrospective self-reports of breastfeeding duration and exclusivity, which introduces measurement error likely to attenuate true associations but could also interact with cohort effects if recall accuracy varies by birth cohort or educational level. Third, the effect sizes are typically small, with differences in IQ or educational attainment that, while statistically significant at the population level, may be of limited clinical relevance for individual children.

Overall, the literature suggests that being breastfed is associated with modest advantages in cognitive performance, educational attainment and some aspects of personality in adulthood. These advantages appear to be partly mediated by higher IQ, which in turn influences educational and occupational trajectories, and may also be shaped by the relational and psychosocial context of breastfeeding. From a policy perspective, these findings reinforce the view that breastfeeding support should be integrated into broader strategies to promote early child development and reduce educational inequalities, while recognising that structural determinants such as poverty, parental education and access to quality early childhood education exert much larger influences on adult human capital than infant feeding alone. Table 4 has all the relevant studies.

## 8. Discussion

This review assessed associations between breastfeeding in infancy and a range of adult health outcomes, including mortality, cardiometabolic health, mental health, cancer risk, cardiovascular disease, and neurodevelopment, within the framework of the DOHaD. Across observational cohorts, quasi-experimental studies and genetic epidemiology, a consistent theme is that breastfeeding contributes to small, directionally favourable shifts in several adult outcomes rather than large changes in individual risk. Based on the results of this review, the clearest associations were observed for cardiometabolic profiles and measures of intellectual and educational performance, whereas findings for adult cancer were modest, heterogeneous, and more difficult to interpret.

For cardiometabolic health, systematic reviews and meta-analyses, birth cohorts from both high- and middle-income settings and large biobank studies converge on a broadly coherent pattern. People who were breastfed as infants tend, on average, to have slightly lower adiposity, more favourable lipid and inflammatory profiles, a lower prevalence of metabolic syndrome and type 2 diabetes, and correspondingly small reductions in cardiovascular disease and all-cause mortality in midlife and older age. These findings are consistent with observational evidence summarised in the WHO review, which reports lower risks of obesity, type 2 diabetes, and hypertension among those who were breastfed. In addition, breastfed adults tend to have lower fasting insulin concentrations and better insulin sensitivity indices independent of body mass index, suggesting potential long-term effects on pancreatic beta-cell function or peripheral insulin action [18].

Evidence from studies in childhood and adolescence indicates a consistent pattern in which breastfeeding is associated with more favourable long-term metabolic health [93,94]. Breastfed infants typically gain weight more slowly after the first months of life and have lower body mass index in later childhood, growth trajectories that are linked to reduced risks of obesity and type 2 diabetes in later life [95,96]. These associations are biologically plausible, given the lower protein content of human milk and the presence of bioactive components that influence appetite regulation, insulin secretion, and energy balance [97]. Early systematic reviews commissioned by the WHO reported a modest reduction in the risk of overweight and obesity in childhood and adolescence among individuals who were breastfed, with pooled odds ratios ranging from 0.78 to 0.86 compared with formula feeding, even after partial adjustment for socioeconomic and perinatal factors [18]. Importantly, quasi-experimental evidence from the PROBIT trial in Belarus, which randomised maternity hospitals to a breastfeeding promotion intervention, strengthens causal inference. This trial demonstrated that longer and more exclusive breastfeeding resulted in small but measurable reductions in childhood adiposity at 6.5 and 11.5 years of age [98]. The consistent associations observed across age groups suggest breastfeeding contributes to a more favourable metabolic risk profile across the life course, acting as an early-life influence that shifts adiposity and metabolic risk in a beneficial direction alongside later lifestyle interventions.

Beyond effects on growth and body composition, breastfeeding may influence later obesity and type 2 diabetes risk through immune and inflammatory pathways, particularly via modulation of the gut microbiome [99]. Human milk provides a rich array of immunologically active and anti-inflammatory components that shape early gut microbial development and reduce systemic inflammation in infants. Key factors—including cytokines (such as IL-6, IL-8, and TGF-β), HMOs, lactoferrin, immunoglobulins, and short-chain fatty acids—selectively promote beneficial microbes like Bifidobacterium and Bacteroides, suppress pathogenic bacteria, and support immune tolerance and intestinal barrier integrity [100,101,102]. Pathway regulation hypotheses further implicate breastfeeding in the regulation of appetite, microbial metabolism, and epigenetic modification of genes involved in energy balance, cardiometabolic regulation, immune function, and neurodevelopment. Emerging data also point to potential roles for breast milk–derived non-coding RNAs, stem cells, and microbial metabolites in mediating long-term metabolic and neurodevelopmental outcomes [103,104,105,106].

Key mechanisms underlying DOHaD include phenotypic plasticity, developmental programming and epigenetic modifications, which mediate the organism’s adaptation to early environmental cues [107,108]. Early nutrition and caregiving can influence DNA methylation, histone modifications, and non-coding RNA expression in genes involved in metabolic regulation, inflammation, and neurodevelopment, with some changes persisting into later life [109]. Emerging evidence suggests that breastfeeding is associated with differential methylation at loci related to lipid metabolism, insulin signalling, and immune regulation, consistent with observed epidemiological links to cardiometabolic and immune outcomes [110]. However, effect sizes across studies are generally small, with risk estimates close to unity, indicating that breastfeeding represents one of many early-life influences on cardiometabolic health and a modest contributor relative to dominant adult risk factors such as smoking, diet, and physical inactivity. Beyond classical endocrine and inflammatory pathways, telomere biology has been proposed as a complementary mechanism linking early-life exposures to later chronic disease susceptibility, through cumulative effects on cellular ageing and inflammatory tone. Recent reviews and emerging empirical work connect cardiometabolic dysregulation with telomere attrition and related pathways, supporting this as a plausible intermediary for life-course risk [111,112,113].

By contrast, current evidence does not indicate a major impact of having been breastfed on overall adult cancer incidence. In the UK Million Women Study, being breastfed in infancy was not associated with overall cancer risk or most major cancer sites after multivariable adjustment, although a modest increase in colorectal cancer was reported [80]. In UK Biobank, being breastfed was associated with only a marginal increase in overall cancer risk in women and no association in men, with site-specific analyses suggesting small elevations for breast and ovarian cancer in women [81]. In the United States, prospective analyses in the Nurses’ Health Study/Nurses’ Health Study II similarly reported a modestly higher adult colorectal cancer risk among participants who had been breastfed, with effect estimates that, while statistically detectable at scale, remain small in absolute magnitude and are not evidence of causation [83]. Evidence from Japan is more limited and includes observational analyses suggesting possible heterogeneity by sex and birth cohort for colorectal cancer, without a clear overall association [84].

Given the small magnitude of reported associations and confidence intervals that often approach unity, chance findings, misclassification of infant-feeding history, and residual confounding remain plausible explanations; therefore, these signals are best regarded as hypotheses requiring replication rather than as established causal effects [80,81,83]. Although several pathways could plausibly link early-life feeding to later cancer risk, including influences on growth trajectories and adiposity, systemic inflammation, gut microbiota development, and immune maturation, the observed patterns, particularly the recurrent signal for colorectal cancer, do not align straightforwardly with the generally favourable cardiometabolic profile associated with breastfeeding, suggesting that any long-term effects (if present) are likely to reflect complex life-course processes rather than direct, uniform causal pathways [80,83].

The evidence for cognitive, educational, and psychological outcomes is more consistent than that for cardiometabolic endpoints. Across the studies we analysed, findings from cohort designs, a randomised breastfeeding promotion trial, and neuroimaging research pointed to small but persistent advantages in cognition and educational attainment associated with longer breastfeeding. While social and family factors may still contribute, the replication of these associations across diverse study designs strengthens the case for a modest causal effect of breastfeeding on neurodevelopment. These conclusions are reinforced by previous long-term follow-up studies, including birth cohorts from Brazil and the PROBIT cluster-randomised trial [114]. Because PROBIT increased breastfeeding duration and exclusivity through a structured promotion intervention rather than maternal self-selection, it provides particularly strong evidence. Children in the intervention group showed modest but consistent improvements in verbal cognition and academic performance, with follow-up at 6.5 and 16 years demonstrating higher verbal IQ scores, better literacy skills, and improved teacher-rated academic achievement [63,114]. These outcomes involve cognitive domains that are strongly predictive of later educational success and mental health. Neuroimaging studies in children and adolescents provide biological plausibility for these long-term associations by identifying structural brain correlates linked to breastfeeding duration. Longer breastfeeding has been associated with differences in cortical thickness, white matter integrity, and regional brain development in networks supporting language, executive function, and socio-emotional processing [88,89,90]. These neural systems underpin not only cognitive performance but also emotional regulation and vulnerability to mental health difficulties, suggesting a pathway through which early feeding experiences may influence psychological outcomes across development.

### Strengths and Limitations

Interpretation of the reviewed evidence is constrained by several methodological limitations. Breastfeeding is socially patterned and correlated with maternal education, income, health behaviours and healthcare access; therefore, residual socioeconomic confounding is plausible even in extensively adjusted analyses. Across the large adult cohorts, breastfeeding exposure was commonly ascertained retrospectively, often decades after infancy, and was usually characterised crudely (e.g., ever versus never breastfed), while linkage to administrative registries for outcomes (mortality, hospitalisation, cancer registration) likely reduced outcome misclassification. Exposure measurement also varied substantially across studies, with limited information in many datasets on exclusivity, duration, mixed feeding, fortification, donor human milk, or neonatal intensive care unit feeding pathways, which increases the risk of non-differential exposure misclassification and may attenuate true associations. Selection processes (including participation and survival to recruitment in midlife cohorts) and differential loss to follow-up may further influence long-term estimates and external validity. Moreover, observed associations for cardiometabolic outcomes were typically small; hazard ratios close to unity should be interpreted as modest shifts in population risk distributions rather than clinically meaningful risk reductions at the individual level. Finally, heterogeneity in mode of birth, prematurity and early morbidity may modify infant-feeding–outcome relations via microbiome, growth and neurodevelopmental pathways, yet these modifiers are inconsistently measured and rarely examined formally across studies.

Notwithstanding these constraints, this review has several strengths. It places breastfeeding within a DOHaD life-course framework, integrates evidence across multiple adult outcome domains, and incorporates genetic epidemiology studies that can help probe causality and reduce confounding. Where available, we emphasised large prospective cohorts with clearly defined outcomes and adjustment for key perinatal and socioeconomic covariates, and we interpreted findings in terms of clinical relevance rather than statistical significance alone. Collectively, the evidence supports breastfeeding promotion for its established early-life benefits and suggests modest, potentially population-relevant shifts in adult cardiometabolic and neurobehavioural profiles, while underscoring the need for triangulation across complementary designs (prospective cohorts, sibling/twin comparisons and Mendelian randomisation) to strengthen causal inference.

## 9. Conclusions

Overall, the evidence to date suggests that breastfeeding in infancy makes a modest but consistent contribution to healthier cardiometabolic profiles and better human-capital outcomes in later life. Adults who were breastfed tend, on average, to have slightly lower adiposity, more favourable metabolic markers, small reductions in cardiovascular disease and mortality, and modest advantages in cognitive performance, education and income. By contrast, any influence on adult cancer risk remains uncertain.

Future research should prioritise contemporary, diverse birth cohorts with prospective, detailed characterisation of infant feeding, coupled with linkage to electronic health records capturing cardiovascular, metabolic, cancer and mental-health outcomes. Integrating epigenetic data, gut microbiota, endocrine profiles and brain structure will be essential to clarify biological pathways. Approaches that explicitly model causal mechanisms and interactions with genetic susceptibility and social context are needed to refine estimates of effect size, to identify subgroups who may benefit most, and to avoid both overstating and underestimating the contribution of breastfeeding to adult health.

From a clinical and public health perspective, breastfeeding should therefore continue to be promoted primarily for its well-established short- and medium-term benefits for infants and mothers, with possible longer-term advantages viewed as an additional benefit rather than the main rationale. It is best considered as one element within a broader life-course strategy for non-communicable disease prevention, not a stand-alone solution.

## Figures and Tables

**Table 1:** Breastfeeding and adult cardiometabolic outcomes and mortality.

Study	Sample/Setting	Design	Adult Outcome(s)	Main Findings
Wang et al., (2023) [65]	UK Biobank; ≈380,000 adults aged 40–73 years	Prospective cohort	All-cause and cause-specific mortality	Being breastfed in infancy was associated with modestly lower all-cause and cardiovascular mortality over ≈12 years of follow-up.
Nakada et al., (2023) [68]	UK Biobank; 320,249 adults aged 40–69	Retrospective cohort (linked hospitalisation/death records)	CVD events/deaths; MI events/deaths	Breastfeeding associated with lower risk of CVD events (HR 0.97), CVD deaths (HR 0.91), MI events (HR 0.93) and MI deaths (HR 0.81) after adjustment
Li et al., (2024) [69]	UK Biobank; ≈360,000 adults aged 40–73 years	Prospective cohort	Incident cardiovascular disease (CHD, stroke, heart failure)	Ever-breastfed participants had slightly lower risks of total CVD and coronary heart disease; associations with stroke and heart failure were weaker.
Zhang et al., (2025) [70]	European adult cohorts; GWAS (total n = 346,821) and a replication dataset (total n = 467,581). GWAS summary statistics for breastfeeding and coronary atherosclerosis	Two-sample Mendelian randomisation (two-step mediation analysis)	Coronary atherosclerosis; HDL-cholesterol as mediator	Genetically proxied breastfeeding in infancy was associated with a lower risk of coronary atherosclerosis, with ≈10% of this protective effect mediated via higher HDL-cholesterol levels
Li et al., (2025) [71]	GWAS summary statistics (2-sample MR); cardiovascular outcomes including CHD, stroke, venous thromboembolism, heart failure, atrial fibrillation/flutter, and type 2 diabetes	Two-sample Mendelian randomisation with multivariable MR and mediation analysis	Major coronary heart disease event; other CVD endpoints; type 2 diabetes; HDL as mediator	Genetically proxied breastfeeding in infancy was causally associated with a lower risk of major CHD. No significant causal associations were observed for venous thromboembolism, stroke, all-cause heart failure, atrial fibrillation/flutter, or type 2 diabetes. Mediation analysis suggested HDL mediated 6.61% of the breastfeeding–CHD effect.

**Table 2:** Breastfeeding and adult adiposity, metabolic syndrome and diabetes.

Study	Sample/Setting	Design	Adult Outcome(s)	Main Findings
Horta et al., (2015) [73]	28 observational studies, many with adult follow-up	Systematic review and meta-analysis	Overweight/obesity, type 2 diabetes, cholesterol, BP	Ever-breastfeeding was associated with ≈13% lower odds of overweight/obesity and ≈35% lower odds of type 2 diabetes; no clear effect on adult BP or cholesterol.
Hu et al., (2025) [75]	UK Biobank; 364,562 adults free of type 2 diabetes at baseline	Prospective cohort	Incident type 2 diabetes; interaction with T2D genetic risk	Ever breastfeeding was associated with a modestly lower 12-year risk of incident T2D, and it attenuated the effect of a T2D genetic risk score (stronger genetic effects and excess risk among never-breastfed participants, particularly those with high genetic risk)
McDade et al., (2025) [74]	Community-based US cohort followed from infancy to early middle adulthood (duration of breastfeeding measured in infancy)	Prospective cohort (life-course observational)	Central adiposity (waist-based measures) and systemic inflammation (e.g., CRP) in early middle adulthood	Longer breastfeeding duration in infancy was associated with lower levels of central adiposity in early middle adulthood and with a more favourable profile of systemic inflammation; associations were most evident for central (abdominal) adiposity and inflammatory biomarkers
Li et al., (2024) [69]	UK Biobank; 364,240 adults aged 40–73 years	Prospective cohort	Incident cardiovascular disease (total CVD, CHD, stroke) and cardiometabolic risk factors	During a median 12.6-year follow-up, being breastfed in infancy was associated with modestly lower risks of total CVD and CHD (weak evidence for stroke), alongside lower adult adiposity, lower CRP, and a lower prevalence of metabolic syndrome.

**Table 3:** Breastfeeding and cancer in adulthood.

Study	Sample/Setting	Design	Adult Outcome(s)	Main Findings
Yang et al. (2019) [80]	Million Women Study, UK; >540,000 women, baseline age 50–64 years	Prospective cohort	Overall and site-specific cancer incidence	Being breastfed in infancy was not clearly associated with overall cancer risk or with most major cancer sites after multivariable adjustment. A modestly higher risk of colorectal cancer, and parallel elevations in benign colorectal polyps and appendicitis, were observed among women who had been breastfed.
Hameiri-Bowen et al., (2024) [81]	UK Biobank; >330,000 adults aged 40–69 years	Prospective cohort	Overall and site-specific cancers	Being breastfed was associated with a very small increase in overall cancer risk in women (HR ≈ 1.05) but not in men. Exploratory analyses suggested slightly higher risks of breast and ovarian cancer in women and a lower risk of oesophageal cancer in men, most of which did not remain significant after correction for multiple testing.
Yuan et al., (2024) [83]	Nurses’ Health Study I and III; ≈159,000 women, >3.5 million person-years	Prospective cohorts	Colorectal cancer and advanced colorectal adenomas	Women who had been breastfed showed a modestly higher risk of colorectal cancer (HR ≈ 1.23) and advanced adenomas, with stronger associations for early-onset disease (<55 years).
Minami et al., (2024) [84]	Japanese multicentre study; >1100 colorectal cancer cases, >1500 benign colorectal tumours, >5000 controls	Case–control nested within a population-based survey	Colorectal cancer and benign colorectal tumours	Overall, no statistically significant association between being breastfed and colorectal cancer or benign tumours after adjustment. Subgroup analyses suggested higher risks in breastfed women born after 1950 and lower risk of benign tumours in breastfed men of the same birth cohort, with wide confidence intervals.

**Table 4:** Breastfeeding and adult cognitive, educational and psychological outcomes.

Study	Sample/Setting	Design	Adult Outcome(s)	Main Findings
Victora et al. (2015) [63]	Pelotas 1982 birth cohort, Brazil; 3493 adults assessed at 30 years	Prospective birth cohort	IQ, years of schooling, income at 30 years	Longer breastfeeding (≥12 months vs. <1 month) was associated with higher IQ, slightly more years of schooling and ~20% higher income at 30 years after multivariable adjustment.
de Mola et al. (2016) [87]	Pelotas 1982 cohort; adults at 30 years	Prospective birth cohort	Adult depression and anxiety symptoms	Being breastfed, particularly for longer durations, was associated with a lower prevalence of common mental disorders in adulthood after adjustment for early-life and socioeconomic factors.
Sutin et al. (2016) [86]	US community sample; ≈9400 adults	Prospective cohort (breastfeeding recalled in adulthood)	Personality traits and lifetime anxiety diagnoses	Adults who had been breastfed scored slightly lower on neuroticism and anxiety and higher on agreeableness and openness; effect sizes were small.
Grevet et al. (2024) [90]	Brazilian High-Risk Cohort for Mental Conditions; 670 children and adolescents with 1326 MRI scans over 8 years	Longitudinal neuroimaging cohort (3 MRI waves, general-ised additive models)	Trajectory of global cortical thickness, cortical area and total intracranial volume from childhood to young adulthood	Longer breastfeeding duration was associated with higher global cortical thickness in both hemispheres and with a more favourable developmental trajectory of total intracranial volume, whereas no association was observed with cortical surface area.
Khudri & Hussey (2025) [85]	US Add Health cohort; ≈13,000 individuals followed into their 30 s	Prospective cohort with econometric models	Educational attainment and employment	Individuals who had been breastfed were more likely to complete secondary and tertiary education; associations with employment status and wages were smaller and less consistent.

## Data Availability

No new data were created or analyzed in this study. Data sharing is not applicable to this article.

## Supplementary Materials

The following supporting information can be downloaded at: https://www.mdpi.com/article/10.3390/children13020286/s1, Table S1: Domain-based appraisal of included key studies (n = 18).

## Abbreviations

The following abbreviations are used in this manuscript:

APA	American Academy of Pediatrics
DNA	Deoxyribonucleic acid
DOHaD	Developmental Origins of Health and Disease
HDL	High-density lipoprotein
HMOs	Human milk oligosaccharides
IQ	Intelligence quotient
NICU	Neonatal Intensive Care Unit
PROBIT	Promotion of Breastfeeding Intervention Trial
RNA	Ribonucleic acid
UK	United Kingdom
US	United States of America
WHO	World Health Organization
