# Supplementary material for: Breastfeeding in Infancy and Adult Health: A Narrative Review
Source: Children (Basel). 2026 Feb 19;13(2):286. doi: 10.3390/children13020286 (PMC12939404; doi:10.3390/children13020286)
Supplement: Supplementary file 1 [file children-13-00286-s001.zip › children-4114673-supplementary.pdf]

Supplementary Table S1. Domain-based appraisal of included key studies (n=18).

Study	Design	Selection / representativeness	Exposure ascertainment	Confounding control	Outcome ascertainment	Attrition / follow-up
**Wang et al. (2023) (1)**	Prospective cohort (UK Biobank)	M	H	M	L	L
**Nakada et al. (2023) (2)**	Linked-record cohort (UK Biobank)	M	H	M	L	L
**Li et al. (2024) (3) [CVD endpoints]**	Prospective cohort (UK Biobank)	M	H	M	L	L
**Zhang et al. (2025) (4)**	Two-sample MR (mediation)	L–M	L	M	L	L
**Li et al. (2025) (5)**	Two-sample MR (MVMR/mediation)	L–M	L	M	L	L
**Horta et al. (2015) (6)**	Systematic review/meta-analysis	M	M–H	M	M	M
**Hu et al. (2025) (7)**	Prospective cohort (UK Biobank)	M	H	M	M–L	L
**McDade et al. (2025)**	Life-course cohort (USA)	M	L	M	M–L	M
**Li et al. (2024) (3) [risk factors]**	Prospective cohort (UK Biobank)	M	H	M	M–L	L
**Yang et al. (2019) (8)**	Prospective cohort (Million Women Study)	M	M	M	L	L
**Hameiri-Bowen et al. (2024) (9)**	Prospective cohort (UK Biobank)	M	H	M	L	L
**Yuan et al. (2024) (10)**	Prospective cohorts (NHS/NHSII)	M	M	M	M–L	M

**Minami et al. (2024) (11)**	Observational (Japan)	M–H	M–H	M–H	M–L	M
**Victora et al. (2015) (12)**	Prospective birth cohort (Pelotas 1982)	M	L	M	M–L	M
**de Mola et al. (2016) (13)**	Prospective birth cohort (Pelotas 1982)	M	L	M	M	M
**Sutin et al. (2016) (14)**	Prospective cohort (USA)	M	M	M	M	M
**Grevet et al. (2024) (15)**	Prospective birth cohort	M	L–M	M	M	M
**Khudri & Hussey (2025) (16)**	Observational cohort (UK/Europe)	M	M–H	M	M	M

Key: L = low concern; M = moderate concern; H = high concern/unclear reporting. Ratings reflect internal validity considerations based on reported methods; where key details were not clearly reported, the domain was rated at least moderate concern.

References

1. Wang X, Yan M, Zhang Y, Wang W, Zhang W, Luo J, et al. Breastfeeding in infancy and mortality in middle and late adulthood: a prospective cohort study and meta-analysis. Journal of internal medicine. 2023;293(5):624-35.
2. Nakada S, Ho FK, Celis-Morales C, Pell JP. Association between being breastfed and cardiovascular disease: a population cohort study of 320 249 participants. Journal of Public Health. 2023;45(3):569-76.
3. Li S, Wang X, Li X, Zhang W, Guo Y, Xu N, et al. Breastfeeding in infancy and cardiovascular disease in middle-aged and older adulthood: a prospective study of 0.36 million UK Biobank participants. The Journal of nutrition, health and aging. 2024;28(10):100347.
4. Zhang B, Yang B, Xiu W, Xue D, Zheng Y, Wei J, et al. HDL cholesterol as a mediator of the relationship between breastfeeding and coronary atherosclerosis from a two-step Mendelian randomization analysis. Scientific Reports. 2025;15(1):28739.
5. Li Q, Zhao C, Lei D, Tong W, Yue C. Causality of breastfed as a baby and cardiovascular disease and the mediating effect of high-density lipoprotein: a Mendelian randomization study. Journal of Dairy Science. 2025.
6. Horta BL, Loret de Mola C, Victora CG. Long-term consequences of breastfeeding on cholesterol, obesity, systolic blood pressure and type 2 diabetes: a systematic review and meta-analysis. Acta paediatrica. 2015;104:30-7.
7. Hu Y, Ma H, Wang X, Zhu S, Chen D, Liang Z. Breastfeeding, genetic susceptibility, and type 2 diabetes in offspring in later life. Obesity. 2025;33(9):1802-9.
8. Yang TO, Cairns BJ, Green J, Reeves GK, Floud S, Bradbury KE, et al. Adult cancer risk in women who were breastfed as infants: large UK prospective study. European journal of epidemiology. 2019;34(9):863.

9. Hameiri-Bowen D, Pedersen DC, Jensen BW, Aarestrup J, Rasmussen KM, Baker JL, et al. The association between being breastfed in infancy and risks of cancer in adulthood—a UK Biobank study. BJC reports. 2024;2(1):40.
10. Yuan C, Wang Q-L, Kim H, Babic A, Zhang J, Wolpin BM, et al. Being breastfed in infancy and risk of colorectal cancer and precursor lesions. Clinical Gastroenterology and Hepatology. 2024;22(7):1508-17. e11.
11. Minami Y, Kanemura S, Kusaka J, Kinouchi M, Suzuki S, Iwasashi H, et al. Association between being breastfed in infancy and adult colorectal cancer risk among Japanese men and women. Scientific Reports. 2024;14(1):9661.
12. Victora CG, Horta BL, De Mola CL, Quevedo L, Pinheiro RT, Gigante DP, et al. Association between breastfeeding and intelligence, educational attainment, and income at 30 years of age: a prospective birth cohort study from Brazil. The lancet global health. 2015;3(4):e199-e205.
13. de Mola CL, Horta BL, Gonçalves H, de Avila Quevedo L, Pinheiro R, Gigante DP, et al. Breastfeeding and mental health in adulthood: A birth cohort study in Brazil. Journal of affective disorders. 2016;202:115-9.
14. Sutin AR, Stephan Y, Terracciano A. Breastfeeding and adult personality. European journal of personality. 2016;30(5):484-91.
15. Grevet LT, Teixeira DS, Pan PM, Jackowski AP, Zugman A, Miguel EC, et al. The association between duration of breastfeeding and the trajectory of brain development from childhood to young adulthood: an 8-year longitudinal study. European Child & Adolescent Psychiatry. 2024;33(6):1863-73.
16. Khudri MM, Hussey A. The long-term impacts of breastfeeding on educational attainment of adults. Review of Economics of the Household. 2025:1-22.
